# Glycogen synthase kinase-3 inhibition attenuates fibroblast activation and development of fibrosis following renal ischemia-reperfusion in mice

**DOI:** 10.1242/dmm.020511

**Published:** 2015-08-01

**Authors:** Shailendra P. Singh, Shixin Tao, Timothy A. Fields, Sydney Webb, Raymond C. Harris, Reena Rao

**Affiliations:** 1 The Kidney Institute, Department of Medicine, University of Kansas Medical Center, Kansas City, KS 66160-3018, USA; 2 Department of Medicine, Vanderbilt University Medical Center, Nashville, TN 37232, USA

**Keywords:** Fibrosis, Glycogen synthase kinase-3β, TGF-β1

## Abstract

Glycogen synthase kinase-3β (GSK3β) is a serine/threonine protein kinase that plays an important role in renal tubular injury and regeneration in acute kidney injury. However, its role in the development of renal fibrosis, often a long-term consequence of acute kidney injury, is unknown. Using a mouse model of renal fibrosis induced by ischemia-reperfusion injury, we demonstrate increased GSK3β expression and activity in fibrotic kidneys, and its presence in myofibroblasts in addition to tubular epithelial cells. Pharmacological inhibition of GSK3 using TDZD-8 starting before or after ischemia-reperfusion significantly suppressed renal fibrosis by reducing the myofibroblast population, collagen-1 and fibronectin deposition, inflammatory cytokines, and macrophage infiltration. GSK3 inhibition *in vivo* reduced TGF-β1, SMAD3 activation and plasminogen activator inhibitor-1 levels. Consistently *in vitro*, TGF-β1 treatment increased GSK3β expression and GSK3 inhibition abolished TGF-β1-induced SMAD3 activation and α-smooth muscle actin (α-SMA) expression in cultured renal fibroblasts. Importantly, overexpression of constitutively active GSK3β stimulated α-SMA expression even in the absence of TGF-β1 treatment. These results suggest that TGF-β regulates GSK3β, which in turn is important for TGF-β–SMAD3 signaling and fibroblast-to-myofibroblast differentiation. Overall, these studies demonstrate that GSK3 could promote renal fibrosis by activation of TGF-β signaling and the use of GSK3 inhibitors might represent a novel therapeutic approach for progressive renal fibrosis that develops as a consequence of acute kidney injury.

## Abstract (precis)

**Summary:** GSK3 promotes renal fibrosis by activation of TGF-β signaling, and the use of GSK3 inhibitors might represent a novel therapeutic approach for progressive renal fibrosis that develops as a consequence of acute kidney injury.

## INTRODUCTION

Renal fibrosis is a classic outcome of multiple forms of chronic kidney disease. It is characterized by excessive extracellular matrix (ECM) remodeling, which results in progressive loss of renal function and often leads to end-stage renal disease (Chuang et al., 2013). Renal fibrosis can also develop as a consequence of maladaptive repair after acute kidney injury (AKI) (Bonventre and Yang, 2011; Venkatachalam et al., 2010). For instance, AKI caused by ischemia-reperfusion (I/R) can result in inadequate tubular regeneration, chronic inflammation, macrophage infiltration and fibroblast activation, leading to excessive ECM deposition and fibrosis (Bonventre and Yang, 2011; Jang et al., 2014; Kim and Padanilam, 2015; Yang et al., 2010; Zhou et al., 2014).

The glycogen synthase kinase-3 (GSK3) family of protein kinases consists of GSK3α and GSK3β isoforms, and plays an important role in injury and repair of renal tubular epithelial cells in AKI. A proapoptotic role for the GSK3β isoform was demonstrated in experimental AKI using gene silencing *in vitro* (Wang et al., 2010), or gene deletion *in vivo* (Howard et al., 2012). Moreover, pharmacological inhibition using isoform non-selective GSK3 inhibitors reduced apoptosis and renal tubular injury in AKI induced by lipopolysaccharides, I/R and nephrotoxins (Bao et al., 2012; Howard et al., 2012; Plotnikov et al., 2013; Wang et al., 2009, 2010). In previous studies we demonstrated that renal-proximal-tubule-specific gene deletion of GSK3β could accelerate renal tubular repair after HgCl_2_-induced AKI in mice (Howard et al., 2012). We also showed that GSK3 inhibition using TDZD-8, 48 h after a nephrotoxic insult, can significantly improve renal tubular repair by increasing pro-proliferative cyclin-D1, c-myc and β-catenin (Howard et al., 2012). These results were subsequently affirmed by studies using LiCl in cisplatin and I/R injury models of AKI (Bao et al., 2014). Thus, inhibition of GSK3 could be a viable strategy for the treatment of AKI. However, it is unclear whether GSK3β is expressed in renal myofibroblasts, the major producers of ECM, or whether GSK3β is involved in the development of renal fibrosis.

GSK3 regulates multiple cell signaling pathways by suppressing accumulation or transcriptional activity of key mediators of these pathways in the absence of ligands or activators (Beurel et al., 2015). Some of these cell-signaling pathways, including TGF-β, Wnt, sonic hedgehog, EGFR and BMP signaling, are important for fibrosis (Chuang et al., 2013; LeBleu et al., 2013). Hence, it could be hypothesized that inhibition of GSK3 would mimic activation of these pro-fibrotic signaling pathways, leading to fibrosis. However, the role of GSK3β in fibrosis seems to be cell- and context-dependent. For instance, *in vitro*, GSK3 inhibition can cause epithelial-to-mesenchymal transition (EMT) in renal tubular epithelial cells by activation of TGF-β1 and an increase in Snail levels (Lan et al., 2014; Noh et al., 2012), whereas, in cultured glomerular mesangial cells, GSK3 inhibition reduces TGF-β1 signaling and fibronectin accumulation (Ho et al., 2012). Moreover, GSK3 inhibition can reduce inflammation, an essential prelude to renal fibrosis (Martin et al., 2005; Wang et al., 2011). Given this complexity and our previous observations that inhibition of GSK3 reduces injury and accelerates repair in the acute setting of AKI, its effect on renal fibrosis, a long-term outcome of AKI, remains to be defined.

In the current studies we examined the role of GSK3β in the development of renal fibrosis and tested the effect of pharmacological inhibition of GSK3 in an I/R-induced mouse model of renal fibrosis. The results of these studies are presented.

**TRANSLATIONAL IMPACT**

**Clinical issue**

Chronic kidney disease affects about 10% of the population and is a major cause of death in the United States and other developed countries. Renal fibrosis – a classic outcome of many forms of chronic kidney disease – is characterized by excessive extracellular matrix (ECM) remodeling, which results in progressive loss of renal function and often leads to end-stage renal disease. Renal fibrosis can also develop as a consequence of maladaptive repair after acute kidney injury (AKI). For instance, AKI caused by ischemia-reperfusion (I/R) can result in inadequate regeneration, chronic inflammation, macrophage infiltration and fibroblast activation, leading to excessive ECM deposition and, in the long-term, renal fibrosis. Glycogen synthase kinase-3β (GSK3β) is a serine/threonine protein kinase that is known to play an important role in injury and regeneration of the renal tubules in AKI. Although inhibition of GSK3β can reduce injury and accelerate repair, its role in the development of renal fibrosis is currently unknown.

****Results****

Here, the authors use a mouse model of renal fibrosis induced by I/R injury to demonstrate that GSK3β expression and activity are increased in fibrotic kidneys and to show that GSK3β is present in myofibroblasts, the major producers of ECM. The authors report that pharmacological inhibition of GSK3 using TDZD-8 (a non-selective inhibitor that acts on both GSK3α and GSK3β) starting before or after I/R injury suppresses renal fibrosis by reducing the myofibroblast population, collagen-1 and fibronectin deposition, the expression of inflammatory cytokines, and macrophage infiltration. GSK3 inhibition *in vivo* reduces *TGF-β1* mRNA levels, SMAD3 activation, and plasminogen activator inhibitor-1 levels. Consistently, TGF-β1 treatment increases GSK3β expression and GSK3 inhibition abolishes TGF-β1-induced SMAD3 activation and α-SMA expression in cultured renal fibroblasts. Importantly, the authors also show that overexpression of constitutively active GSK3β stimulates α-SMA expression even in the absence of TGF-β1 treatment.

****Implications and future directions****

These results indicate that, after I/R injury, TGF-β regulates renal GSK3β, which in turn is important for TGF-β–SMAD3 signaling and fibroblast-to-myofibroblast differentiation. Thus, GSK3 could promote renal fibrosis after AKI by activation of TGF-β signaling. The finding that GSK3 inhibition, starting even after AKI has occurred, can reduce fibrosis is important because a large percentage of AKI cases are detected only after fibrosis has begun to develop. The use of GSK3 inhibitors might, therefore, represent a novel approach for the treatment of the progressive renal fibrosis that develops as a consequence of AKI.

## RESULTS

### Renal GSK3β expression increases following I/R

**Fig. 1.**
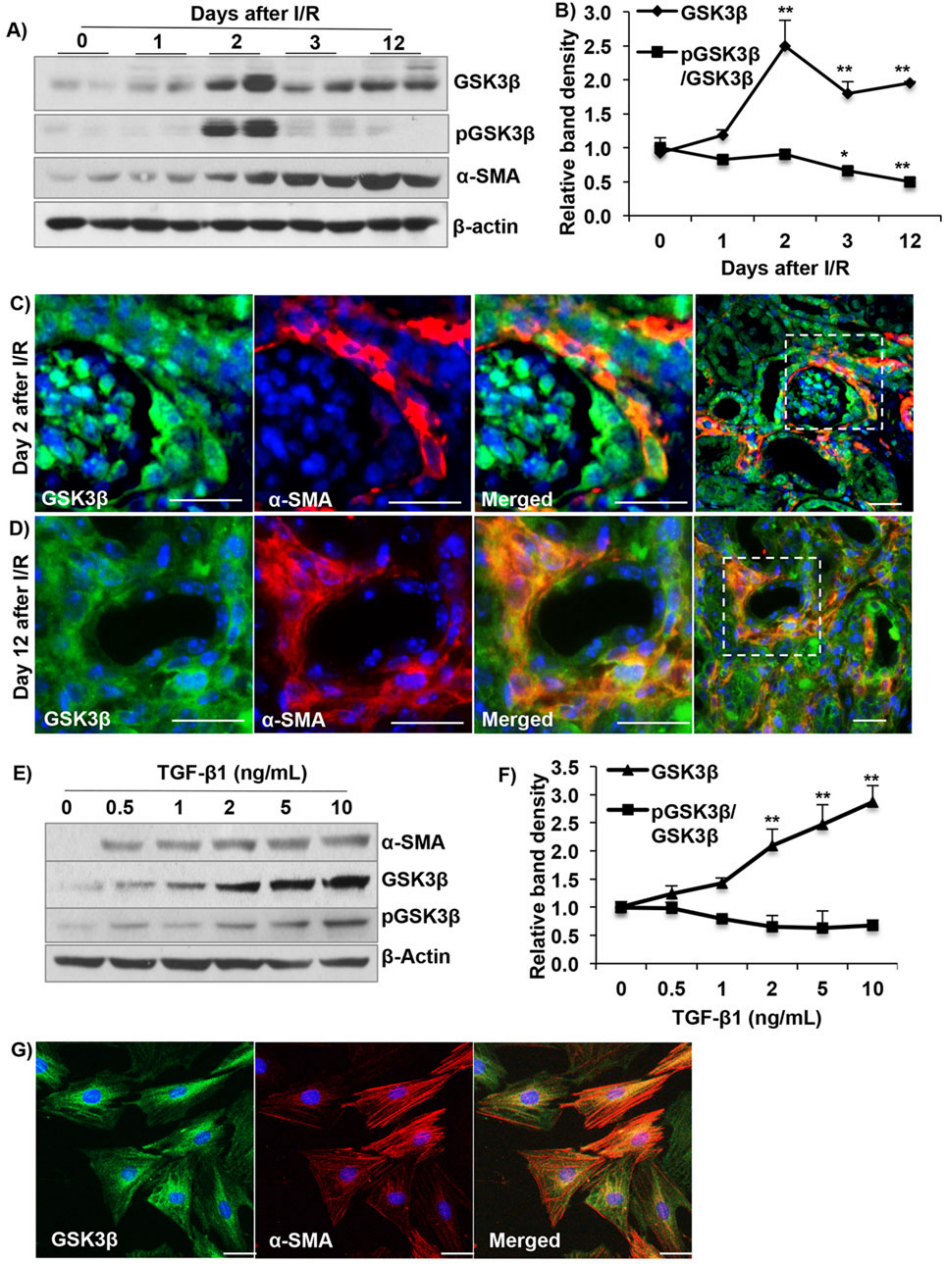
**Increased GSK3β expression after renal I/R.** (A) Western blot analysis and (B) quantitation of band density show increased GSK3β protein levels and reduced inactive pGSK3β-serine-9/GSK3β ratio and increased α-SMA in kidneys after I/R. *P<0.05, **P<0.01 compared to day-0. (C,D) GSK3β (green) and α-SMA (red) co-staining shows that GSK3β is expressed in renal myofibroblasts on (C) day-2 and (D) day-12 after I/R. Scale bars: 25 µm. (E) In rat renal fibroblast-NRK-49F cells, TGF-β1 treatment for 48 h dose-dependently increased GSK3β and pGSK3β levels. (F) Relative band densities of total GSK3β and pGSK3β/GSK3β ratio. **P<0.01, compared to 0 ng/ml TGF-β1 treatment. (G) GSK3β colocalizes with α-SMA in NRK-49F cells. Scale bars: 25 µm. *n*=6 mice/group for immunoblotting and *n*=4 mice/group for immunostaining.

To determine the role of GSK3β in the development of renal fibrosis, we first examined its expression and activation in the kidneys of mice subjected to bilateral renal I/R. A time-course analysis of renal GSK3β expression following I/R showed a significant increase in total GSK3β levels by day-2, which at day-12 remained twofold higher than at day-0 (Fig. 1A,B). The serine-9 phosphorylated (inactive) form of GSK3β (pGSK3β) increased significantly by day-2, following which it returned to baseline levels. The ratio of pGSK3β to GSK3β did not change significantly on day-2 and was further reduced on day-3 and -12, suggesting an increase in GSK3β activity (Fig. 1A,B). Expression levels of renal α-smooth muscle actin (α-SMA), a marker of myofibroblasts, also increased, starting on day-2 following I/R (Fig. 1A). Immunofluorescence (IF) staining demonstrated that GSK3β colocalizes with α-SMA in day-2 as well as day-12 I/R kidneys (Fig. 1C). The day-12 I/R kidneys were fibrotic as determined by Masson's-trichrome staining and Sirius-red staining (supplementary material Fig. S1A). GSK3β expression was detected in proximal tubules and, to a lower extent, in collecting ducts, but not thick ascending limbs (supplementary material Fig. S1B). Unlike proximal tubules and myofibroblasts, macrophages (stained by the marker F4/80) in day-12 I/R kidneys rarely stained for GSK3β (supplementary material Fig. S1C).

To determine whether TGF-β1, a known stimulator of fibroblast-to-myofibroblasts differentiation, can stimulate GSK3β expression, immortalized rat fibroblasts (NRK-49F cells) were treated with TGF-β1. Treatment with TGF-β1 dose-dependently increased GSK3β as well as α-SMA. GSK3β expression doubled at a dose of 2 ng/ml of TGF-β1 and colocalized with α-SMA in myofibroblasts (Fig. 1E-G), whereas the ratio of pGSK3β to GSK3β remained unchanged (Fig. 1E,F). These results demonstrate for the first time increased and sustained GSK3β expression in fibrotic kidneys following I/R or following TGF-β1 treatment *in vitro*, and its association with myofibroblasts.

### Inhibition of GSK3 attenuates development of renal fibrosis after I/R

**Fig. 2.**
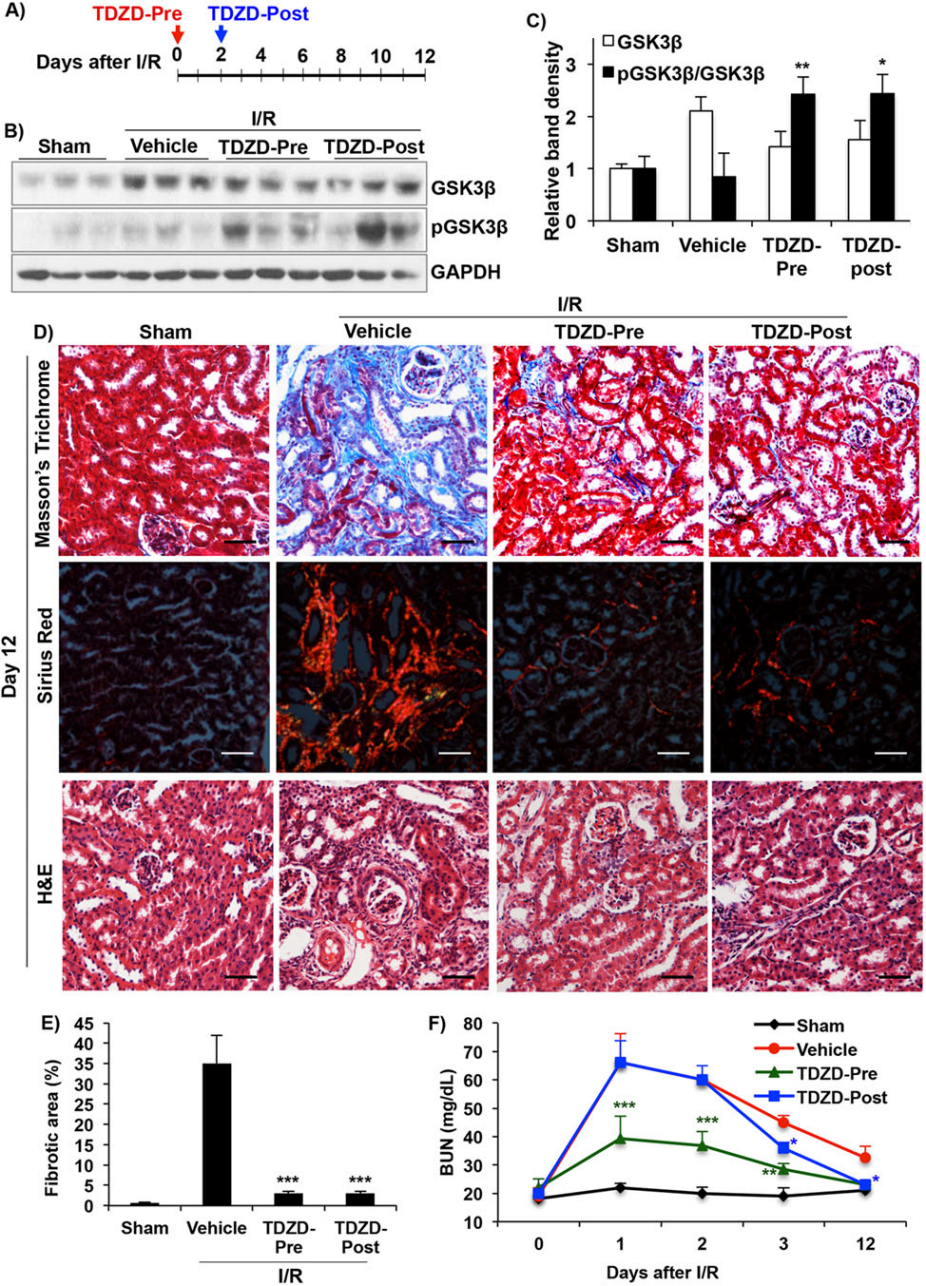
**Treatment with TDZD, a GSK3 inhibitor, reduced fibrosis following I/R.** (A) Scheme of experimental design. Treatment with TDZD (1 mg/kg BWt), starting 1 hour before I/R (TDZD-Pre) and 2 days after I/R (TDZD-Post). (B) Western blot analysis and (C) densitometry shows increased pGSK3β/GSK3β ratios in TDZD-treated kidneys. (D) Masson's trichrome-, Picro-sirius red- and H&E-stained kidney sections show reduced injury and fibrosis in the TDZD-Pre and TDZD-Post treatment groups 12 days after I/R. (E) Fibrosis score based on Masson's trichrome staining. (F) Blood urea nitrogen (BUN) levels. *P<0.05, **P<0.01, ***P<0.001 compared to vehicle-treated I/R group, *n*=6 mice/group. Scale bars: 50 µm. All tissue samples were from day-12 after I/R.

To determine the role of GSK3 activity in I/R-induced renal fibrosis, we examined the effect of TDZD-8 (TDZD) (Martinez et al., 2002), a highly specific ATP non-competitive inhibitor of GSK3 that we and others have effectively used in mice (Howard et al., 2012; Tao et al., 2015; Wang et al., 2010). Because GSK3β expression increased by day-2 (Fig. 1A), TDZD treatment [1 mg/kg body weight (BWt)] was started on day-2 after I/R in one group of mice (TDZD-Post) and, in a second group, 1 h before I/R (TDZD-Pre) (Fig. 2A). All mice were sacrificed on day-12 after I/R. TDZD treatment did not affect renal GSK3β levels, but significantly increased pGSK3β and pGSK3β/GSK3β levels when compared to vehicle-treated I/R kidneys, suggesting GSK3β inhibition (Fig. 2B,C). As illustrated by trichrome and Sirius-red staining, the TDZD-Pre and TDZD-Post, I/R groups showed a striking reduction in fibrosis compared to the vehicle-treated I/R group (Fig. 2D,E).

Dilated tubules and tubular atrophy were observed in the vehicle-treated I/R group by H&E staining (Fig. 2D), whereas the TDZD-Pre and TDZD-Post treatment groups showed minimal injury. Following I/R, blood urea nitrogen (BUN) levels in the TDZD-Pre treatment group never increased to the high levels as in the vehicle treated group (Fig. 2F). Similarly in the TDZD-Post treatment group, BUN levels reduced significantly as early as 24 h after initiation of treatment (day-3) and decreased to TDZD-Pre treatment levels by day-12. These results indicate that GSK3 inhibition not only preserves renal function in AKI, but TDZD treatment, either pre- or post-I/R, can reduce fibrosis.

### TDZD treatment inhibits renal ECM deposition and reduces the myofibroblast population

**Fig. 3.**
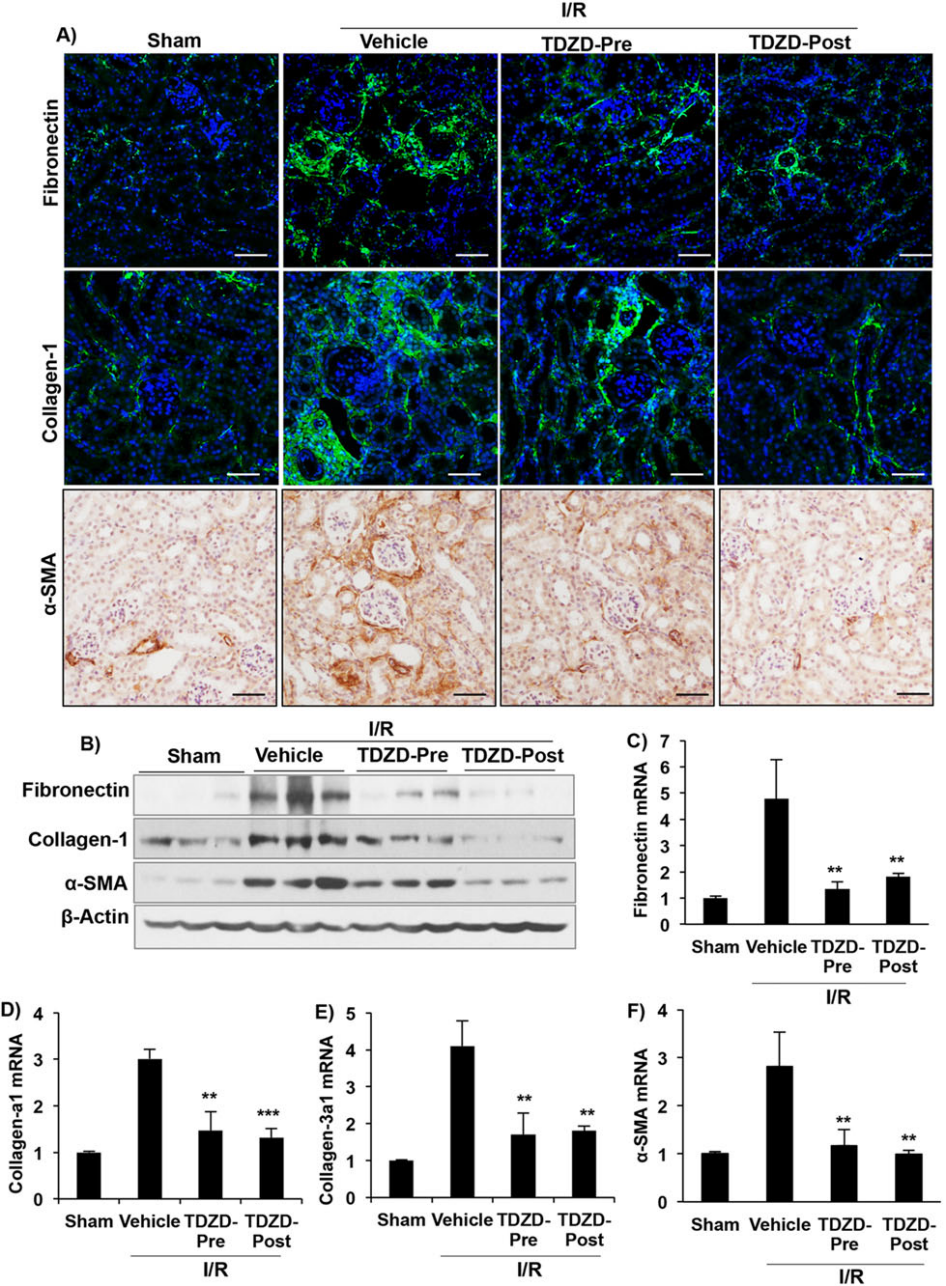
**GSK3 inhibition reduced ECM deposition following I/R.** (A) Immunostaining and (B) western blot for fibronectin, collagen-1 and α-SMA show reduced levels in the TDZD-Pre and TDZD-Post treatment groups. (C) Quantitative RT-PCR to determine mRNA levels of fibronectin, (D) collagen-a1, (E) collagen 3a1 and (F) *α-SMA* (fold change, relative to β-actin). **P<0.01, ***P<0.001 compared to vehicle-treated I/R group, *n*=6 mice/group. Scale bars: 50 µm.

To determine the effect of GSK3 inhibition on ECM deposition, we measured expression levels of collagen-1 and fibronectin, major matrix components. Because active myofibroblasts are a major source of collagen and fibronectin, we also determined the myofibroblast population in the kidneys by measuring α-SMA expression. Immunostaining for fibronectin, collagen-1 and α-SMA was higher in vehicle-treated I/R kidneys compared to sham kidneys, and TDZD treatment reduced their expression (Fig. 3A). Western blot analysis confirmed these results and showed that, compared to TDZD-Pre, the TDZD-Post treatment group had significantly lower levels of collagen-1, fibronectin and α-SMA (Fig. 3B, supplementary material Fig. S2). Fibronectin, collagen-a1, collagen-3a1 and *α-SMA* mRNA levels were also increased in vehicle-treated I/R kidneys compared to sham and significantly reduced in TDZD treatment groups (Fig. 3C-F), although no significant difference was observed in mRNA levels between the TDZD-Pre and TDZD-Post treatment groups. These results suggest that inhibition of GSK3 activity can reduce the myofibroblast population and ECM deposition following I/R-injury-induced fibrosis.

### GSK3 inhibition reduced proinflammatory factors and macrophage infiltration

**Fig. 4.**
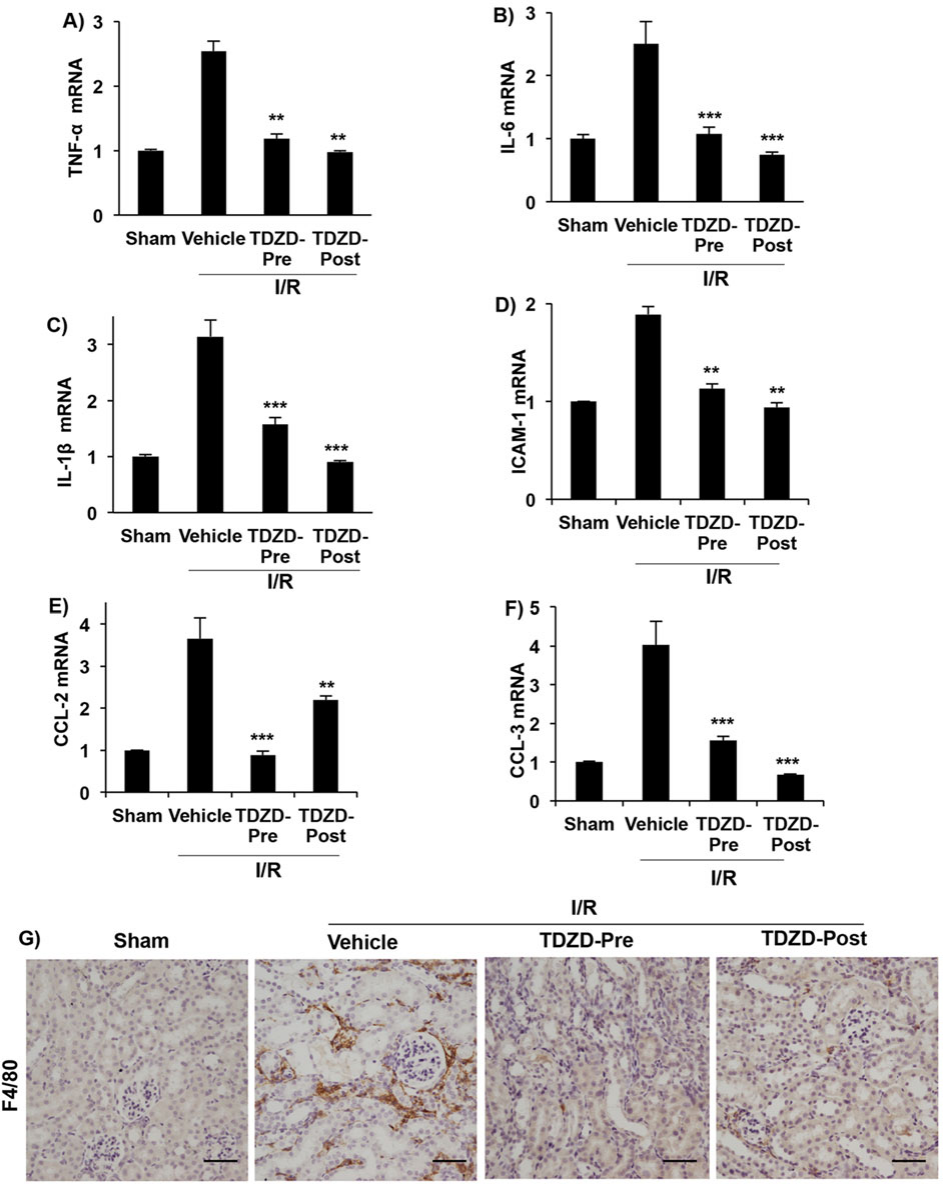
**GSK3 inhibition reduced pro-inflammatory cytokines and macrophage infiltration following I/R.** mRNA levels of (A) *TNF-α*, (B) *IL-6*, (C) *IL-1β*, (D) *ICAM-1*, (E) *CCL-2* and (F) *CCL-3* (fold change, relative to β-actin) are lower in TDZD-treated than vehicle-treated I/R kidneys. (G) F4/80 staining shows reduced macrophage infiltration in TDZD-treated compared to vehicle-treated I/R kidneys. **P<0.01, ***P<0.001 compared to vehicle-treated I/R group, *n*=6 mice/group. Scale bars: 50 µm.

Fibroblast activation can be stimulated by pro-inflammatory cytokines, chemokines and growth factors secreted by damaged epithelial cells, myofibroblasts and infiltrating cells that further enhance tissue fibrosis. We found significant upregulation of mRNA encoding the cytokines TNF-α, IL-6 and IL-1β in vehicle-treated I/R kidneys, compared to sham (Fig. 4A-C). mRNA levels of ICAM-1, a myofibroblast-expressed mediator for interaction with infiltrating leukocytes, were also increased in vehicle-treated I/R kidneys (Fig. 4D). Similarly, monocyte chemoattractants CCL-2 (Furuichi et al., 2009) and CCL-3 (Correa-Costa et al., 2014) were significantly increased in vehicle-treated I/R kidneys (Fig. 4E,F), accompanied by increased macrophage infiltration determined by immunostaining for F4/80 (Fig. 4G). These proinflammatory factors and macrophage infiltration were significantly reduced in the TDZD-Pre and TDZD-Post treatment groups. These results suggest that GSK3 activity could contribute to macrophage infiltration and the production of proinflammatory cytokines in I/R-induced fibrosis.

### GSK3 inhibition blocks TGF-β signaling but increases β-catenin in epithelial cells

**Fig. 5.**
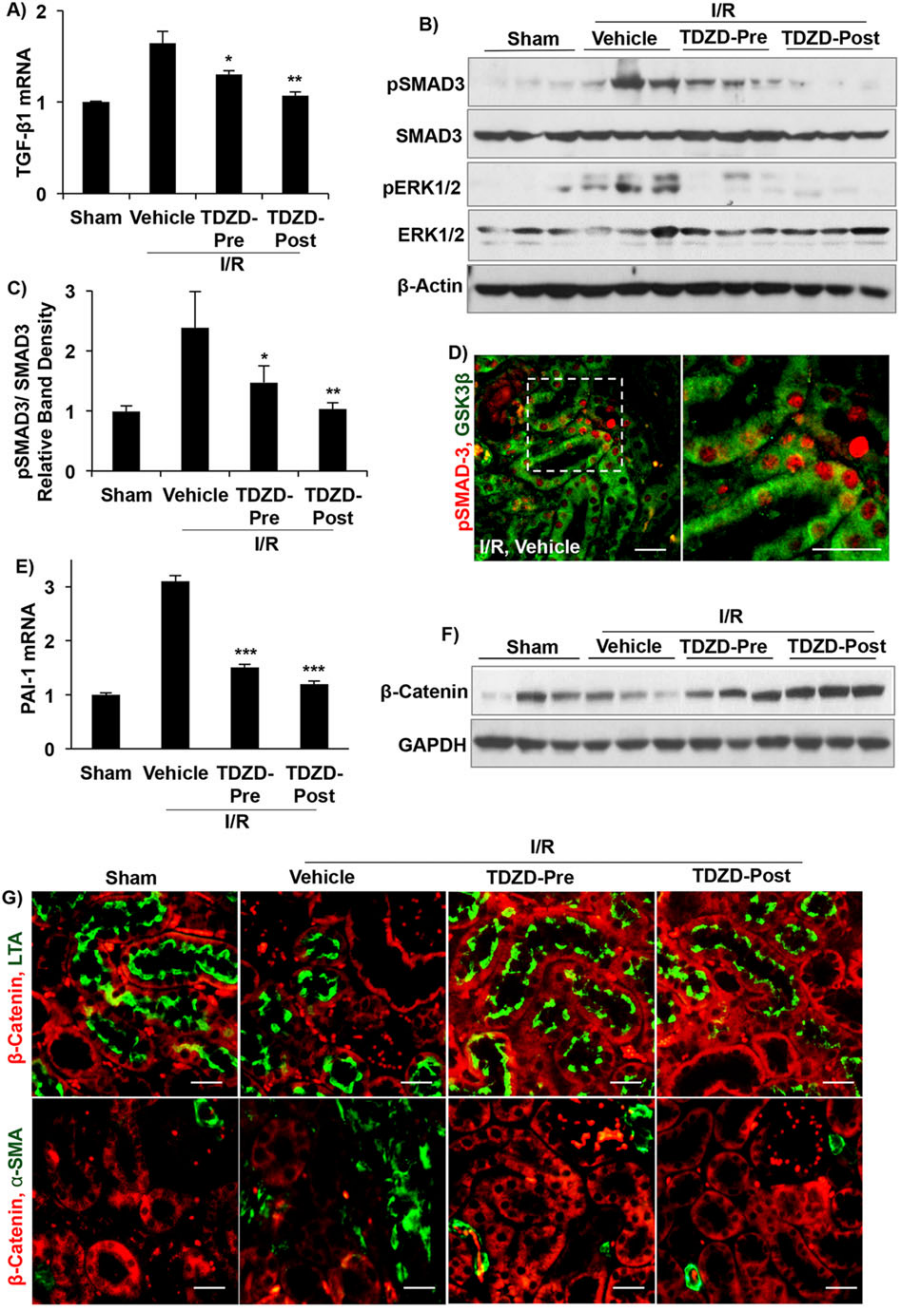
**GSK3 inhibition reduced TGF-β signaling after I/R.** (A) Quantitative RT-PCR shows reduced *TGF-β1* mRNA in TDZD-treated compared with vehicle-treated I/R kidneys (fold change, relative to β-actin). (B) Western blot analysis for pSMAD3, SMAD3, pERK1/2 and ERK1/2, and (C) pSMAD3/SMAD3 ratio based on relative band density. (D) Immunostaining for pSMAD3 (red) and GSK3β (green). (E) Quantitative RT-PCR for *PAI-1*. (F) Western blot analysis shows increased β-catenin levels in TDZD-treated mouse kidney compared to sham- or vehicle-treated kidneys. (G) Immunostaining for β-catenin (red), LTA (green) or α-SMA (green). *P<0.5, **P<0.01, ***P<0.001 compared to vehicle treated I/R group, *n*=6 mice/group. Scale bars: 25 µm.

TGF-β signaling plays a crucial role in the development of renal fibrosis. Hence, we examined the effect of TDZD treatment on canonical TGF-β signaling. *TGF-β1* mRNA levels were 60% higher in the vehicle-treated I/R group compared to sham (Fig. 5A), with a corresponding increase in TGF-β signaling, suggested by increased pERK and pSMAD3 levels (Fig. 5B,C). IF staining demonstrated pSMAD3 expression in GSK3β-expressing cells (Fig. 5D). In TDZD-Pre and TDZD-Post I/R kidneys, *TGF-β1* mRNA, pERK and pSMAD levels were significantly reduced compared to vehicle-treated I/R kidneys (Fig. 5A-C). mRNA levels of TGF-β receptor 1 and 2, and *CTGF* were unchanged in the TDZD treatment groups compared to vehicle treatment group (data not shown). PAI-1, an inhibitor of collagen degradation, is encoded by an important gene that is upregulated by TGF-β signaling (Ma and Fogo, 2009; Samarakoon et al., 2012). In vehicle-treated I/R kidneys, *PAI-1* mRNA levels were threefold higher compared to sham, and TDZD treatment significantly reduced *PAI-1* mRNA levels (Fig. 5E).

Canonical Wnt signaling regulates expression of multiple pro-inflammatory and pro-fibrotic factors (DiRocco et al., 2013). We examined β-catenin, an important component of Wnt signaling, to determine whether TDZD treatment increased its stabilization. Indeed, in TDZD-Pre and TDZD-Post I/R kidneys, β-catenin protein levels were significantly higher than in sham- or vehicle-treated I/R kidneys (Fig. 5F, supplementary material Fig. S3). However, β-catenin expression in TDZD-Pre and TDZD-Post I/R kidneys could be detected only in renal tubules, including *Lotus tetragonolobus* agglutinin (LTA)-staining proximal tubules (Fig. 5G), but not in the few α-SMA expressing myofibroblasts (Fig. 5G). These results suggest that inhibition of GSK3 suppressed renal fibrosis following I/R by inhibiting TGF-β signaling and not β-catenin-dependent mechanisms.

### GSK3 is crucial for TGF-β-induced fibroblast-to-myofibroblast differentiation *in vitro*

**Fig. 6.**
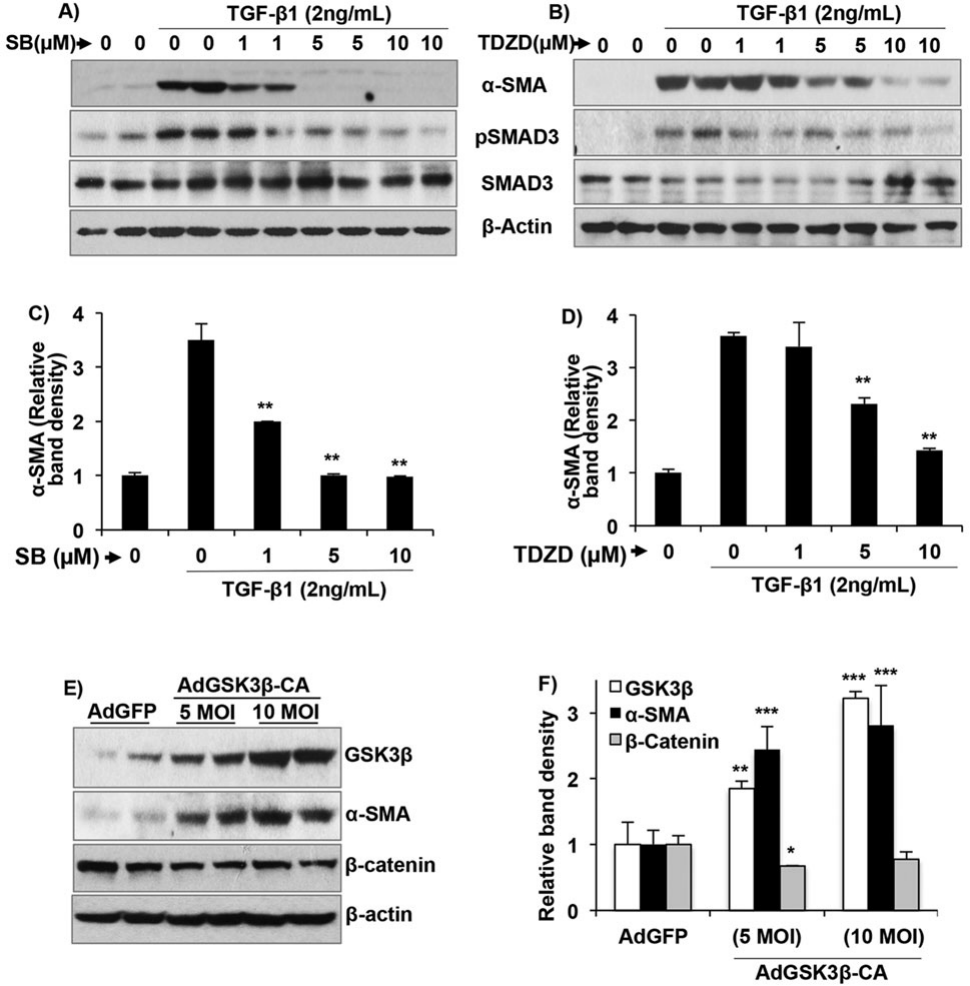
**GSK3 activity is critical for TGF-β1-induced fibroblast activation.** Inhibition of GSK3 using (A) SB216763 (SB) or (B) TDZD dose-dependently reduced α-SMA expression and pSMAD3 levels in NRK-49F cells treated with TGF-β1 for 48 h. (C,D) Quantitation of relative band density for α-SMA. (E) Overexpression of constitutively active GSK3β-serine 9-alanine (GSK3β-CA) in NRK-49F cells increases α-SMA but reduces β-catenin compared to cells overexpressing GFP (control). (F) Quantitation of relative band density. *P<0.5, **P<0.01, ***P<0.001 compared to control, *n*=4.

To further determine the role of GSK3 in myofibroblasts, we tested the effect of the GSK3 inhibitors TDZD and SB216763 (a small-molecule inhibitor of GSK3) on TGF-β1-induced α-SMA expression in NRK-49F cells. Pre-treatment with SB216763 (Fig. 6A,C) or TDZD (Fig. 6B,D) significantly reduced α-SMA expression in a dose-dependent fashion. The GSK3 inhibitors also reduced TGF-β1-induced pSMAD3 levels (Fig. 6A,B). To further determine whether an increase in GSK3β in renal fibroblasts can stimulate α-SMA expression, we overexpressed constitutively active GSK3β (AdGSK3β-CA, serine 9-alanine mutant) or control GFP (AdGFP) in NRK-49F cells. After 48 h, GSK3β expression increased in the AdGSK3β-CA transduced cells, accompanied by an increase in α-SMA expression and decrease in β-catenin levels (Fig. 6E,F). These studies demonstrate that GSK3β plays a crucial role in TGF-β signaling and fibroblast activation.

## DISCUSSION

Renal fibrosis is characterized by activation and proliferation of fibroblasts, which continually produce and deposit ECM proteins, leading to progressive fibrosis. Herein we demonstrate that GSK3β is expressed in myofibroblasts, and GSK3β expression and activity are increased in mouse kidneys following I/R and in cultured fibroblasts following TGF-β1 treatment. Pharmacological inhibition of GSK3 using TDZD significantly reduced pro-inflammatory and pro-fibrotic cytokines, macrophage infiltration and ECM deposition, thereby reducing fibrosis. GSK3 inhibition reduced the myofibroblast population *in vivo* and fibroblast-to-myofibroblast differentiation *in vitro* by a TGF-β–SMAD signaling-dependent mechanism. Thus, GSK3β plays a pro-fibrotic role in the kidney following I/R, and its inhibition, even after the injury has occurred, could prevent the future development of fibrosis.

GSK3β is expressed in proximal tubules (Nørregaard et al., 2015), and increased GSK3β has been detected in renal tubules of human chronic renal allograft dysfunction tissue by immunohistochemical staining (Gong et al., 2008; Yan et al., 2012). In the current study we found a time-dependent and sustained increase in GSK3β expression and activity following I/R in mouse kidneys. Importantly, and unlike the previous studies, GSK3β expression was detected in α-SMA-expressing myofibroblasts, as early as day-2 following I/R. Moreover, increased GSK3β expression could be linked to fibroblast activation because overexpression of constitutively active GSK3β (serine 21 to alanine mutation) by itself led to an increase in α-SMA expression similar to the effect of TGF-β1 treatment in NRK-49F cells.

Fibroblast activation/differentiation into myofibroblasts, which produce large amounts of ECM components, is a key step in the development of fibrosis (Kalluri and Zeisberg, 2006; LeBleu et al., 2013; Strutz and Zeisberg, 2006). In the vehicle-treated I/R kidneys, we found a large population of myofibroblasts, which was significantly reduced by TDZD treatment. Fibroblasts can be activated by paracrine and autocrine factors, including pro-inflammatory and pro-fibrotic cytokines and chemokines produced by injured tubular epithelial cells, macrophages and myofibroblasts themselves (Grande and Lopez-Novoa, 2009; Kendall and Feghali-Bostwick, 2014). In I/R kidneys, a significant increase in IL-6, IL-1β, TNFα, TGF-β1, macrophage chemoattractants and infiltration of macrophages was found, which were abolished by TDZD treatment. These results are consistent with a pro-inflammatory role for GSK3 (Dugo et al., 2007; Gong et al., 2008; Martin et al., 2005) and suggest that suppressed production of one or more of these cytokines/chemokines could result in reduced fibroblast activation in the TDZD-treated mice.

The chemokine TGF-β, which promotes fibroblast activation, proliferation, migration and ECM synthesis, is a critical mediator of fibrosis (Kramann et al., 2013; Zarjou and Agarwal, 2012). Canonical TGF-β signaling involves binding of TGF-β to its receptors and activation of SMAD3, which, together with SMAD4, regulates the expression of pro-fibrogenic genes (Farris and Colvin, 2012). The role of GSK3 in TGF-β signaling is controversial. In *in vitro* studies, GSK3 inhibition by pharmacological inhibitors or activation of Wnt signaling attenuated TGF-β1-mediated ECM accumulation in cultured renal glomerular mesenchymal cells, lung, and gingival and skin fibroblasts (Bahammam et al., 2013; Ho et al., 2012; Liu et al., 2012). Similarly, in cultured human renal tubular epithelial cells and corneal fibroblasts, GSK3 inhibition reduced TGF-β1-induced SMAD3 activity (Choi et al., 2011; Zhang et al., 2007). On the other hand, TGF-β inhibits GSK3β via ERK-MAPK in hepatocellular carcinoma (Ding et al., 2005) and cultured peritoneal mesothelial cells (Jang et al., 2013), and GSK3 inhibition can lead to SMAD3 activation and fibrosis in cultured cardiac myocytes and fibroblasts (Hua et al., 2010; Lal et al., 2014). In the kidney, GSK3 inhibition has been associated with EMT in unilateral ureteral obstruction (UUO) *in vivo* and renal epithelial cells *in vitro* by increasing TGF-β1-induced β-catenin and Snail accumulation (Lan et al., 2014; Noh et al., 2012; Yoshino et al., 2007; Zhou et al., 2004). Moreover, in a transgenic mouse expressing constitutively active GSK3α and GSKβ, α-SMA levels did not increase following 3 days of UUO (Voelkl et al., 2013). The results of the current studies in an I/R model of AKI are consistent with a pro-fibrotic role for GSK3β by its involvement in TGF-β signaling in the kidney. We demonstrate that pSMAD3 expression colocalized with GSK3β in fibrotic kidney following I/R and systemic GSK3 inhibition significantly reduced expression of *TGF-β1*, pSMAD3 and *PAI-1*, a SMAD-regulated profibrotic gene. This suggests that TGF-β–SMAD3 signaling is active in vehicle-treated I/R kidneys and GSK3 inhibition suppresses it. Moreover, GSK3 inhibition using TDZD or SB216763 abolished TGF-β1-induced SMAD3 activation and α-SMA expression in cultured renal fibroblasts. Because TGF-β1 treatment also increased GSK3β expression and activity, GSK3β could play an essential role in TGF-β1-signaling-mediated fibroblast activation and development of fibrosis.

In addition to SMAD, the Wnt signaling pathway also mediates the effects of TGF-β1 (Akhmetshina et al., 2012; Zhou, 2011). Wnt signaling has been implicated in the pathogenesis of renal fibrosis based on the findings that expression of Wnt ligands is upregulated in fibrotic kidneys, and inhibition of Wnt signaling using DKK-1, sFRP4 or paricalcitol can reduce renal fibrosis in mice (DiRocco et al., 2013; Hao et al., 2011; He et al., 2009; Ren et al., 2013; Surendran et al., 2005). GSK3 is linked to canonical Wnt signaling by virtue of its ability to prevent cytoplasmic accumulation of β-catenin in the absence of Wnt ligands (Kaidanovich-Beilin and Woodgett, 2011). In the absence of Wnt ligands, GSK3β phosphorylates β-catenin, which prevents its cytoplasmic accumulation (Yost et al., 1996). In the presence of Wnt ligands, GSK3β is unable to phosphorylate β-catenin, leading to its accumulation and increased activity. Although the role of Wnt ligands in the development of renal fibrosis is clear, the role of β-catenin, an important component of canonical Wnt signaling, has been inconclusive. Renal β-catenin is increased in experimental models of renal fibrosis (He et al., 2009; Ren et al., 2013; Surendran et al., 2005) and its systemic inhibition can reduce fibrosis (Hao et al., 2011). However, although DKK-1, a Wnt antagonist significantly reduced renal β-catenin abundance (He et al., 2009), the anti-fibrotic effects of DKK-1 were found to be independent of β-catenin in UUO and I/R models of fibrosis (Ren et al., 2013). Moreover, gene deletion of β-catenin in tubular epithelium did not reduce renal fibrosis (Zhou et al., 2013). In the current studies, renal β-catenin abundance increased significantly in TDZD-treated mice, consistent with our previous studies (Howard et al., 2012). However, β-catenin expression in TDZD-treated mice was localized to tubules and, consistent with Zhou et al.'s observation (Zhou et al., 2013), increased renal tubular β-catenin levels did not result in increased α-SMA, ECM or fibrosis. Regardless of the role of β-catenin in the development of renal fibrosis, the involvement of GSK3 in fibroblast activation or renal fibrosis does not seem to be linked to β-catenin in the post-I/R kidney. This is further supported by the findings that both increased β-catenin (von Toerne et al., 2009) and increased GSK3β (Gong et al., 2008; Yan et al., 2012) occur in renal tubules of human chronic renal allograft nephropathy.

Injured tubules are known to recruit macrophages, secrete cytokines and chemokines, and generally induce fibrosis (Bonventre and Yang, 2011). Because GSK3β expression was observed in both renal tubular epithelial cells and interstitial fibroblasts, it cannot be excluded at this point that GSK3 inhibition could have led to complete and proper repair of the renal tubules after I/R and thereby resulted in reduced inflammation, TGF-β signaling and fibrosis. However, the *in vitro* and *in vivo* findings that (a) GSK3β is expressed in myofibroblasts, (b) GSK3β activity is crucial for TGF-β signaling and α-SMA expression, and (c) constitutively active GSK3β can increase α-SMA expression *in vitro*, suggest that GSK3β could play an important role in fibroblast differentiation and renal fibrosis.

In summary, our study is the first to demonstrate that GSK3 is a key pathogenic determinant in the development of renal fibrosis. The pro-fibrogenic role of GSK3 in the post I/R kidney could be coupled to TGF-β signaling, although additional studies are needed to address the mechanism. As such, the results show that pharmacological inhibition of GSK3, even after the detection of AKI in patients, could suppress fibroblast activation and development of renal fibrosis.

## MATERIALS AND METHODS

### Bilateral I/R surgery and experimental protocol

Bilateral I/R was carried out essentially as described earlier (Wei and Dong, 2012) on male C57/BL6J mice (Jackson Laboratory, Bar Harbor, MN) weighing approximately 25 g. Briefly, both renal pedicles were exposed by flank incisions and clamped using micro aneurysm clamps for 30 min under pentobarbital anesthesia [60 mg/kg BWt, intraperitoneal (IP)]. At the end of the ischemic period, the clamps were released for reperfusion.

Study groups were: (1) sham: mice underwent surgery to expose renal pedicle, without clamping; (2) vehicle-treated I/R: mice underwent surgery for I/R and received vehicle injection (10% DMSO) 1 h before clamping; (3) TDZD-Pre I/R: mice underwent surgery for I/R and received TDZD daily, starting 1 h before I/R; (4) TDZD-Post I/R: mice underwent surgery for I/R and received TDZD daily, starting 48 h after I/R. *n*=6 for each group.

TDZD (TDZD-8) was dissolved in 10% DMSO and administered by daily IP injection at a dose of 1 mg/kg BWt. All experiments were approved by the IACUC committee of University of Kansas Medical Center. Blood was collected from tail vein and plasma used to measure BUN using a QuantiChrom Urea Assay Kit from BioAssay Systems (Hayward, CA) following the manufacturer's instructions.

### Quantitative real-time PCR (RT-PCR)

Total RNA was extracted using TRIzol (Sigma-Aldrich, MO) and first-strand cDNA synthesized using a reverse transcription system kit (Applied Biosystems, NY). RT-PCR was performed on an ABI PRISM 7000 sequence detection system (Applied Biosystems, Foster City, CA). Primer sequences are provided in supplementary data Table S1. The mRNA levels were calculated relative to β-actin levels for each sample.

### *In vitro* studies

NRK-49F cells (ATCC, VA) were cultured in DMEM medium containing 5% fetal bovine serum, 0.5% penicillin and streptomycin. Cells were serum starved for 16 h followed by TGF-β1 (Sigma-Aldrich, MO) treatment for 48 h. Adenovirus*,* AdGSK3β-CA, carrying a serine-to-alanine substitution at Ser-9 in the NH2-terminal region of GSK3β was a gift from Dr Thomas Force (Haq et al., 2000). AdGSK3β-CA and control, AdGFP, have been described before (Rao et al., 2004). The recombinant viruses were propagated in HEK 293 cells, and high titer stocks (2×10^10^ particles/ml) were purified by CsCl density gradient centrifugation. For infection of NRK-49F cells, virus of 5 or 10 multiplicity of infection (MOI) was added to each culture dish.

### Western blot analysis

Kidney tissues were lysed in RIPA buffer and loaded onto SDS-PAGE gels, transferred to nitrocellulose membranes and blocked with 5% milk in TBST. Membranes were probed with primary antibody followed by TBST washes and horseradish peroxidase secondary antibody application. Secondary antibodies were purchased from Dako (CA).

### Antibodies used

For western blot and immunostaining of tissue sections, antibodies for GSK3β, pGSK3β, β-catenin, fibronectin, α-SMA (Cell Signaling Technology, Inc., MA), collagen I (MD Bioproducts, MN), GAPDH, pERK and ERK (Santa Cruz Biotechnology, Inc., TX), β-actin (Sigma-Aldrich, MO) were used. Secondary antibodies were purchased from Dako (CA). Antibodies used only for immunohistochemistry (IHC)/IF staining were anti-α-SMA, F4/80 (Serotec, NC), PCNA (Dako, CA), DBA and LTA (Vector Laboratories, CA), BrdU (Cell Signaling Technology, Inc., MA), and pSMAD3 (Rockland, PA).

### Histology, immunohistochemistry and immunofluorescence

Kidney sections were fixed in 4% paraformaldehyde and blocked in paraffin. Picro sirius red staining (Polysciences, PA) was carried out and fibrillar collagen was visualized under polarized light. Masson's trichrome staining was carried out using staining kit (Polysciences, PA). For both IHC and IF, paraffin sections were de-paraffinised, washed in PBS containing 0.1% Tween 20 (PBST) and blocked in 10% normal goat serum.

Primary antibodies were applied to sections and incubated at 4°C overnight. For IHC, slides were blocked with Avidin/Biotin (Invitrogen, NY), and then biotinylated goat anti-rabbit IgG or anti-mouse IgG (Invitrogen, NY) secondary antibodies were applied, followed by incubation with Streptavidin HRP conjugate (Invitrogen, NY). Finally, slides were developed with DAB (Vector Laboratories, CA) and counterstained with Harris Haematoxylin, dehydrated, and mounted with Permount (Fisher Scientific). For IF, after incubation with primary antibody, goat anti-rabbit IgG Alexa-Fluor-488, goat anti-mouse IgG Texas red, or goat anti-chicken IgG Alexa-Fluor-555 secondary antibodies were applied, and, following incubation, washed, stained with DAPI and mounted with Flour-G (Invitrogen, NY). All images were captured using a Nikon 80i microscope in KUMC imaging center.

### Statistics

Values are expressed as mean±standard error for all bar charts, except for band density measurements of western blots, which is expressed as mean±standard deviation. Data was analyzed using Graphpad Prism software (Version 5.0d). Two-tailed unpaired *t*-test with Welch's correction and *F*-test to compare variances and One-Way Analysis of Variance followed by Tukey's multiple comparison test and Bartlett's test for equal variance were used. A probability level of 0.05 (*P*≤0.05) was considered significant.

## Supplementary Material

Supplementary Material
