# Supplementary material for: Glycogen synthase kinase-3 inhibition attenuates fibroblast activation and development of fibrosis following renal ischemia-reperfusion in mice
Source: Dis Model Mech. 2015 Aug 1;8(8):931–40. doi: 10.1242/dmm.020511 (PMC4527294; doi:10.1242/dmm.020511)
Supplement: Supplementary Material [file supp_8_8_931__index.html]

Supplementary Material 

# Glycogen synthase kinase-3 inhibition attenuates fibroblast activation and development of fibrosis following renal ischemia/reperfusion in mice

## DMM020511 Supplementary Material

- Supplementary Material
