# Supplementary material for: Glycogen synthase kinase-3 inhibition attenuates fibroblast activation and development of fibrosis following renal ischemia-reperfusion in mice
Source: Dis Model Mech. 2015 Aug 1;8(8):931–40. doi: 10.1242/dmm.020511 (PMC4527294; doi:10.1242/dmm.020511)
Supplement: Supplementary Material [file supp_020511_DMM020511supp.pdf]

Supplemental Figures

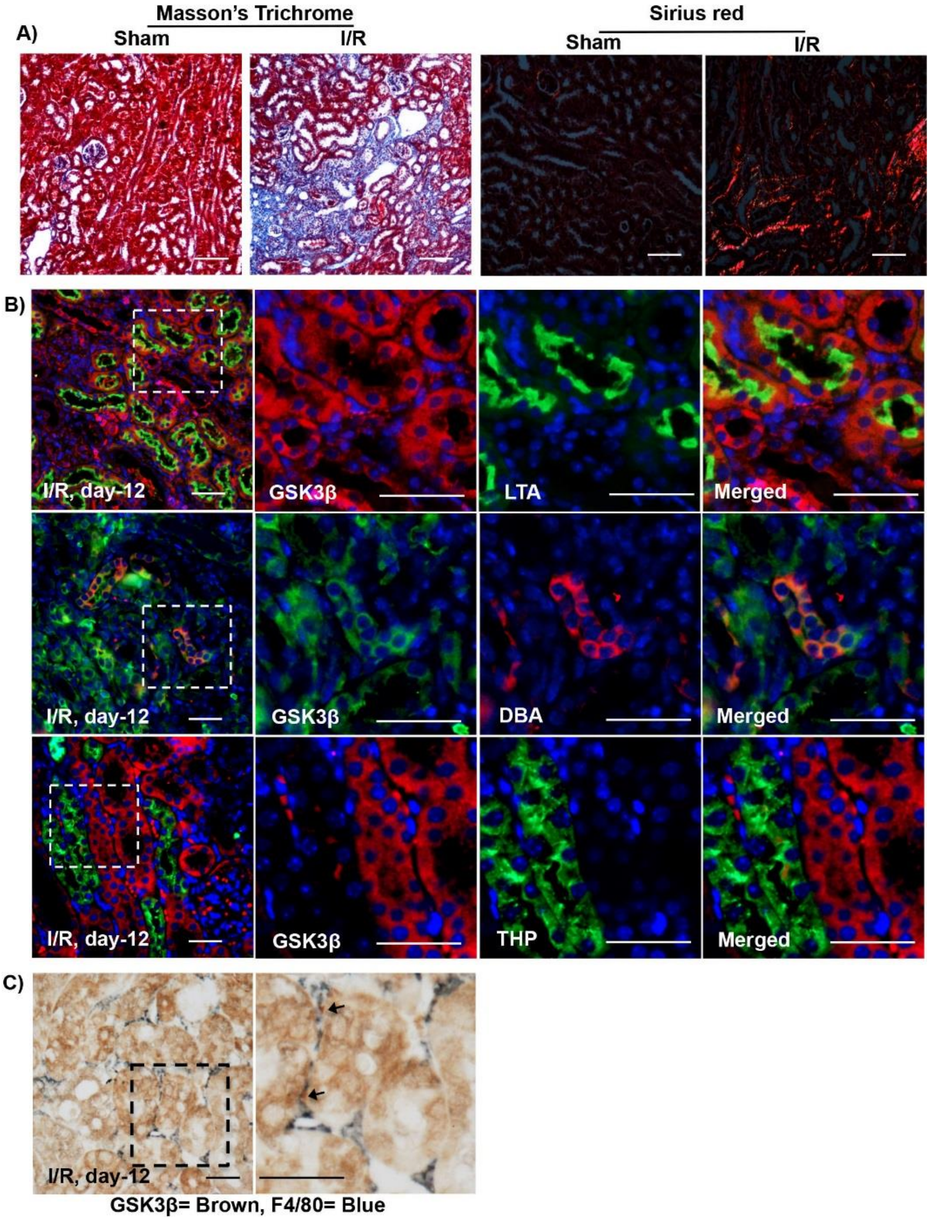

Supplemental 1:

(A) Masson's-trichrome staining and Sirius red staining shows fibrosis in mouse kidneys 12 days after I/R. Masson's trichrome staining, scale bar=100µm, Sirius red staining, scale bar=50µM.

(B) GSK3β (red) and *Lotus tetragonolobus* agglutinin (LTA, green for proximal tubule), GSK3β (green) and *Dolichos biflorus* agglutinin (DBA, red for collecting duct) and GSK3β (red) and Tamm-Horsfall protein (THP, green for thick ascending limb). (Scale bar=50µm).

(C) GSK3β (Brown) F4/80 (blue) staining representing macrophages (Scale bar=25µm).

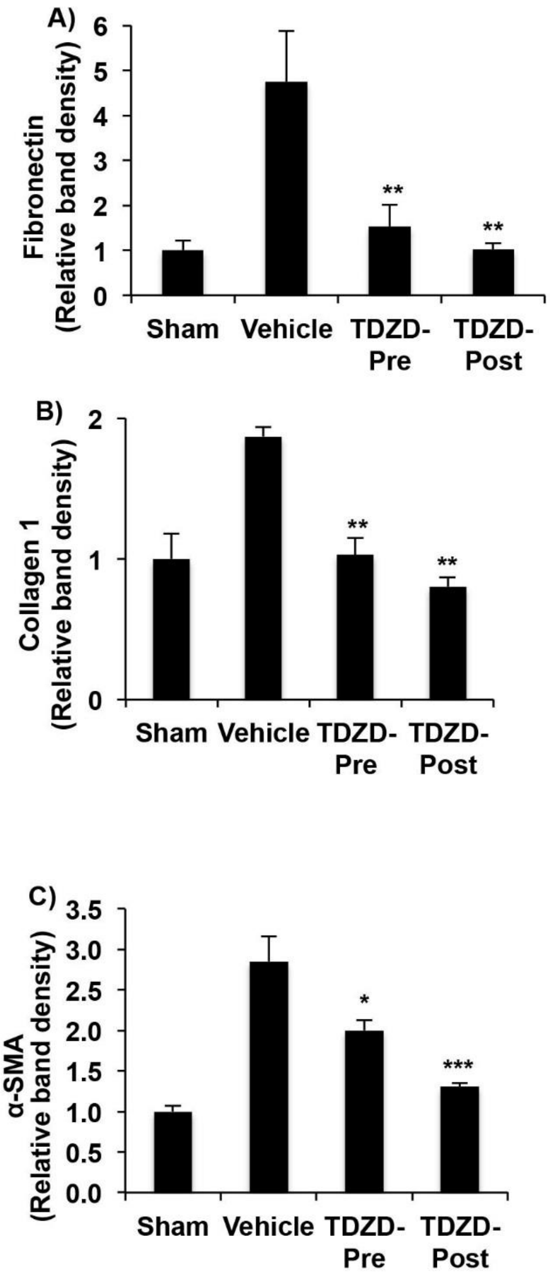

Supplemental-2: Quantitation of band density of Western blot from Fig 3b shows reduced (A) fibronectin, (B) collagen-1 and (C) α-SMA in TDZD treated I/R kidneys compared to vehicle treated I/R kidneys. *P<0.05, **P<0.01, ***P<0.001 compared to vehicle treated I/R kidneys.

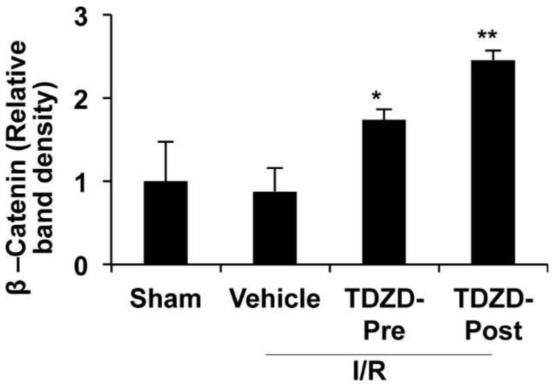

Supplemental-3: Quantitation of band density of Western blot from Fig 5F shows increased β-catenin levels in TDZD treated mouse kidney compared to sham or vehicle treated kidneys. *P<0.05, **P<0.01, compared to vehicle treated I/R kidneys.

Supplemental- Table 1:

Primers used for RTPCR:

	Gene Name	Primers
1	Fibronectin	F ATGTGGACCCCTCCTGATAGT<br>R GCCCAGTGATTTCAGCAAAGG
2	Collagen-1a1	F AGA CAT GTT CAG CTT TGT GGAC<br>R GCA GCT GAC TTC AGG GAT G
3	Collagen-3a1	F TCC CCT GGA ATC TGT GAA TC<br>R TGA GTC GAA TTG GGG AGA AT
4	FSP-1	F TGTAATTGTGTCCACCTTCC<br>R GCTCATCACCTTCTGGAATG
5	α-SMA	F TCAGGGAGTAATGGTTGGAATG<br>R GGTGATGATGCCGTGTTCTA
6	TNF-α	F ACC CTC ACA CTC AGA TCA TCT TC<br>R TGG TGG TTT GCT ACG ACG T
7	IL-1β	F TTG ACG GAC CCC AAA AGA T<br>R GAA GCT GGA TGC TCT CAT CTG
8	ICAM-1	F CTTCCAGCTACCATCCCAAA<br>R CTTCAGAGGCAGGAAACAGG
9	Ccl2	F CTCGGACTGTGATGCCTTAAT<br>R TGGATCCACACCTTGCATTTA
10	Ccl3	F GAAGATTCCACGCCAATTCATC<br>R GATCTGCCGGTTTCTCTTAGTC
11	TGF-β1	F TGAGTGGCTGTCTTTTGACG<br>R AGCCCTGTATTCCGTCTCCT
12	TGF-βRI	F CTGGGCAAAGATTAGGGTGA<br>R GCTGGCCACTACTTCTGAGG
13	TGF-βRII	F ACAGGGGTCACACAGGACTC<br>R GCTCATTCCCTGCTCTCATC
14	TGF-βRIII	F ATGGTCCCCTGTGTAGCTTG<br>R GCGGAGTATCAGGAGTCAGC
15	PAI-1	F GAGGTGGAAAGAGCCAGATTTA<br>R CCACTGAAGTAGAGGGCATTC
